# Association between COVID-19 Burden, Population Vaccination Status, and Urologic Oncology Surgery Volume: A National Multicenter Cross-Sectional Study

**DOI:** 10.3390/jcm11237071

**Published:** 2022-11-29

**Authors:** Ido Amir, Shay Golan, Michael Frumer, Itay A. Sternberg, Omri Schwarztuch Gildor, Azik Hoffman, Reut Shashar, Roy Mano, Ziv Savin, Miki Haifler, Dorit E. Zilberman, Zohar A. Dotan, Barak Rosenzweig

**Affiliations:** 1 Sackler Faculty of Medicine, Tel Aviv University, Tel Aviv 6997801, Israel; 2 Section of Urology, Rabin Medical Center, Petah Tikva 4941492, Israel; 3 Israeli Urologic Oncology Collaboration (IUOC);; 4 Department of Urology, Meir Medical Center, Kfar Saba 4428164, Israel; 5 Department of Urology, Rambam Health Center, Haifa 3109601, Israel; 6 Ruth and Bruce Rappaport Faculty of Medicine, The Technion-Israel Institute of Technology, Haifa 3200003, Israel; 7 Department of Urology, Tel Aviv Sourasky Medical Center, Tel Aviv 6423906, Israel; 8 Department of Urology, Chaim Sheba Medical Center, Ramat Gan 5262000, Israel

**Keywords:** COVID-19, surgical oncology, urologic-oncology, vaccination campaign

## Abstract

Initial deleterious effects of the COVID-19 pandemic on urologic oncology surgeries are well described, but the possible influence of vaccination efforts and those of pandemic conditions on surgical volumes is unclear. Our aim was to examine the association between changing vaccination status and COVID-19 burden throughout the pandemic and the volume of urologic oncology surgeries in Israel. This multi-center cross-sectional study included data collected from five tertiary centers between January 2019 and December 2021. All 7327 urologic oncology surgeries were included. Epidemiological data were obtained from the Israeli Ministry of Health database. A rising trend in total urologic oncology surgery volumes was observed with ensuing COVID-19 wave peaks over time ($X^{2}$ = 13.184, *df* = 3, *p* = 0.004). Total monthly surgical volumes correlated with total monthly hospitalizations due to COVID-19 (*R* = −0.36, *p* = 0.015), as well as with the monthly average Oxford Stringency Index (*R* = −0.31, *p* = 0.035). The cumulative percent of vaccinations and of new COVID-19 cases per month did not correlate with total monthly urologic surgery volumes. Our study demonstrates the gradual acclimation of the Israeli healthcare system to the COVID-19 pandemic. However, hospitalizations due to COVID-19, as well as restriction stringency, correlate with lower volumes of urologic oncological surgeries, regardless of the population’s vaccination status.

## 1. Introduction

Since its designation as a global pandemic by the World Health Organization in March 2020, coronavirus disease 2019 (COVID-19) has had a profound impact on healthcare systems worldwide [1]. Government responses were variously comprised of strict containment measures, including travel restrictions, economic lockdowns, and stringent health-oriented measures [2]. While these measures affected patient populations in various aspects of care [3], they especially exacted a toll on oncologic patients who had a 3.5-fold higher susceptibility to severe COVID-19-related events compared to patients without cancer [4]. Significant delays in cancer-related surgeries have been documented globally, leading to decreased survival rates [5,6,7,8]. Several coping strategies have been proposed, including updated guidelines regarding diagnostic and therapeutic modalities, triaging, and risk-stratification models of oncologic diseases [8,9]. For instance, the European Association of Urology (EAU) had formulated adapted clinical guidelines considering the effects of the COVID-19 pandemic on patient care. Diagnosis, treatment, and follow-up recommendations were classified according to anticipated potential clinical harm; Levels of priority include low (clinical harm very unlikely if postponed for 6 months), intermediate (clinical harm possible if postponed for 3 months, but unlikely), high (clinical harm very likely if postponed for >6 weeks) and emergency (cannot be postponed for >24 hours) [10]. Data on the effect of such measures on surgical volumes, however, are lacking. As of December 2020, the Pfizer BNT162b2 mRNA vaccine has been available for use in Israel, the first nation to administer a COVID-19 vaccine on a large scale, and the first to administer booster shots [11,12]. To the best of our knowledge, no study to date had examined the impact of vaccination campaigns and the rapidly evolving pandemic response policies on oncologic surgery volumes. In this report, we aimed to use the Israeli healthcare system as a model for the potential positive effects of COVID-19 vaccination campaigns on surgical volumes of patients diagnosed with urologic malignancies.

## 2. Materials and Methods

### 2.1. Participants

A total of 7327 urologic oncology surgeries undertaken between January 2019 and December 2021 were included in our analysis. The data were retrieved from five Israeli tertiary medical centers, including Sheba Medical Center, Rabin Medical Center, Tel Aviv Sourasky Medical Center, Rambam Medical Center, and Meir Medical Center. Data collection was by ICD-10 codes for adrenalectomy, nephroureterectomy, partial and radical nephrectomy, radical cystectomy, radical orchiectomy, radical prostatectomy, retroperitoneal lymph node dissection (RPLND), and transurethral resection of bladder tumor (TURBT). No data on patient demographics were included in our study.

### 2.2. Quantification of COVID-19 Restrictions

We used the calculated Oxford Stringency Index (OSI) for assessing the effect of the Israeli government’s containment measures on surgical volumes [2]. Calculated by the Oxford Coronavirus Government Response Tracker (OxCGRT) project, the stringency index is a composite measure of nine distinct response metrics; These include eight policy indicators of containment and closure policies, as well as one health system policy indicator (presence of public information campaigns). The stringency index reports a numeric score ranging between 0 and 100, with a higher score reflecting stricter measures [13]. A monthly average of the OSI was calculated since surgical volumes were reported on a monthly basis. For the sake of convenience, OSI values were classified into three distinct categories as proposed by Glasbey et al. [14]: light restrictions (<20), moderate lockdowns (20–60), and full lockdowns (>60).

### 2.3. National Vaccination Rates

Since vaccination rates varied for each national vaccination campaign, with the highest compliance recorded during the initial campaign in December 2020, and for simplicity of reporting, our analysis limited the assessment of rates to the first dose. It is important to note, however, that the study period also includes the second dose and the booster shots provided nationwide.

### 2.4. “COVID-19 Wave” Definition

To date, there is no standard and widely accepted definition of a “COVID-19 wave” [15]. We arbitrarily opted to include a two-month period in each wave and only the time periods that reflected a rising trend in COVID-19 cases with a subsequent peak, as reflected in the Israeli Ministry of Health database [16], yielding a total of four COVID-19 waves for inclusion into the study.

### 2.5. Statistical Analysis

Correlations between monthly surgical volumes and the COVID-19 parameters of new COVID-19 cases, new COVID-19-related hospitalizations, average monthly OSI findings, and the cumulative first-dose vaccination percentage were analyzed with the Spearman’s rank correlation coefficient. A subgroup analysis by surgery type was also performed. Chi-squared values were calculated for goodness-of-fit. An alpha value of 5% defined significance, and all tests were one-tailed according to relevant hypotheses. Data analysis and plotting were performed with R version 4.0.5.

### 2.6. Institutional Review Board (IRB) Approvals

Appropriate IRB approvals were granted by each participating medical center.

## 3. Results

### 3.1. Patient Characteristics

**Figure 1:**
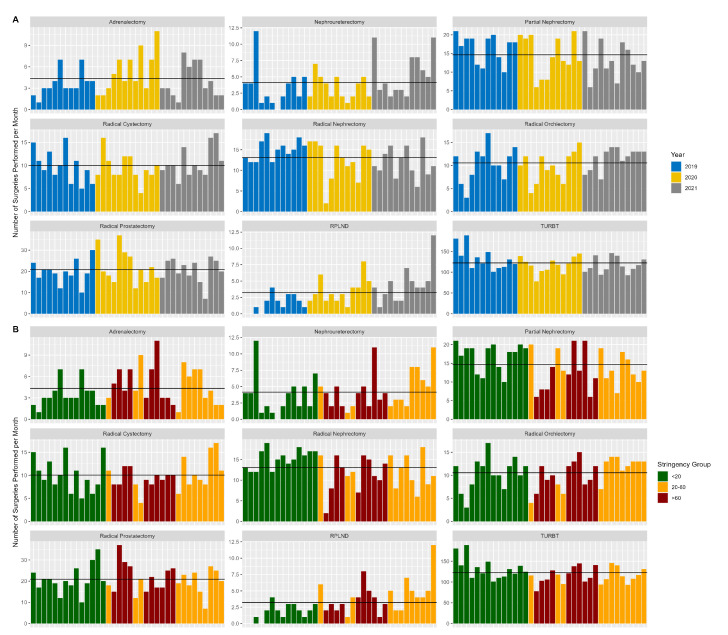
(**A**) Monthly urologic surgery volumes per year and subgroup analysis by surgery type. (**B**) Monthly surgical volumes per stringency category and subgroup analysis by surgery type. The horizontal black line indicates the overall mean number of surgeries.

Data were collected on the number of adrenalectomies (*n* = 156), nephroureterectomies (*n* = 149), partial nephrectomies (*n* = 528), radical nephrectomies (*n* = 471), radical cystectomies (*n* = 361), radical orchiectomies (*n* = 380), radical prostatectomies (*n* = 751), RPLND procedures (*n* = 116), and TURBTs (*n* = 4415) that were performed in the five participating medical centers between 1 January 2019 and 31 December 2021. Changes in monthly surgical volumes per year and per stringency category are shown in Figure 1.

### 3.2. Correlations between New COVID-19 Cases in Israel and Monthly Urologic Oncology Surgical Volumes

**Figure 2:**
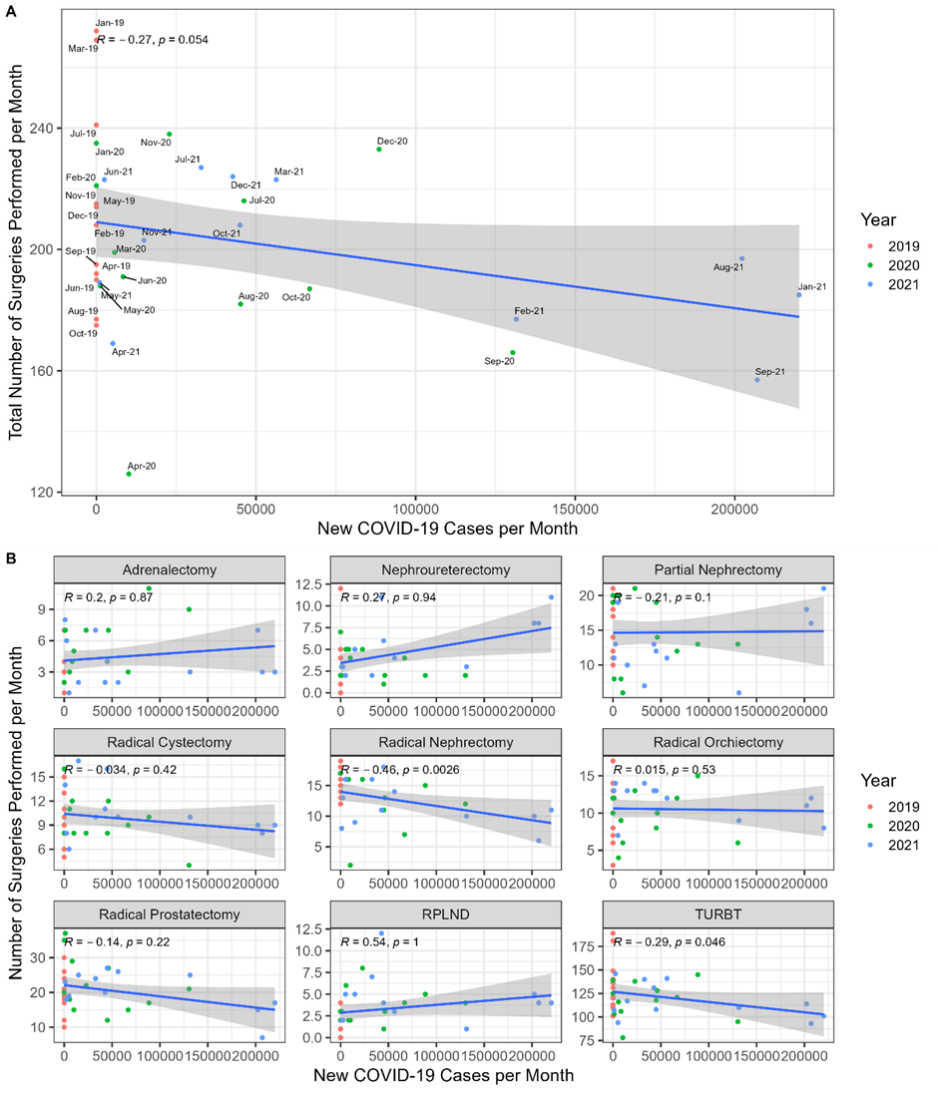
(**A**) Correlation between the total number of urologic surgeries per month and the number of new COVID-19 cases per month. (**B**) Subgroup analysis per surgery type.

We hypothesized that greater numbers of COVID-19 cases would have a negative effect on urologic oncology surgical volumes. Interestingly, there was no significant correlation between the total number of surgeries performed per month and the number of new COVID-19 cases, but rather a possible trend (*R* = −0.27, *p* = 0.054) (Figure 2). A subgroup analysis per surgery type demonstrated a significant negative correlation for radical nephrectomies (*R* = −0.46, *p* = 0.0026) and TURBTs (*R* = −0.29, *p* = 0.046).

### 3.3. Correlations between New COVID-19 Related Hospitalizations in Israel and Monthly Urologic Oncology Surgical Volumes

**Figure 3:**
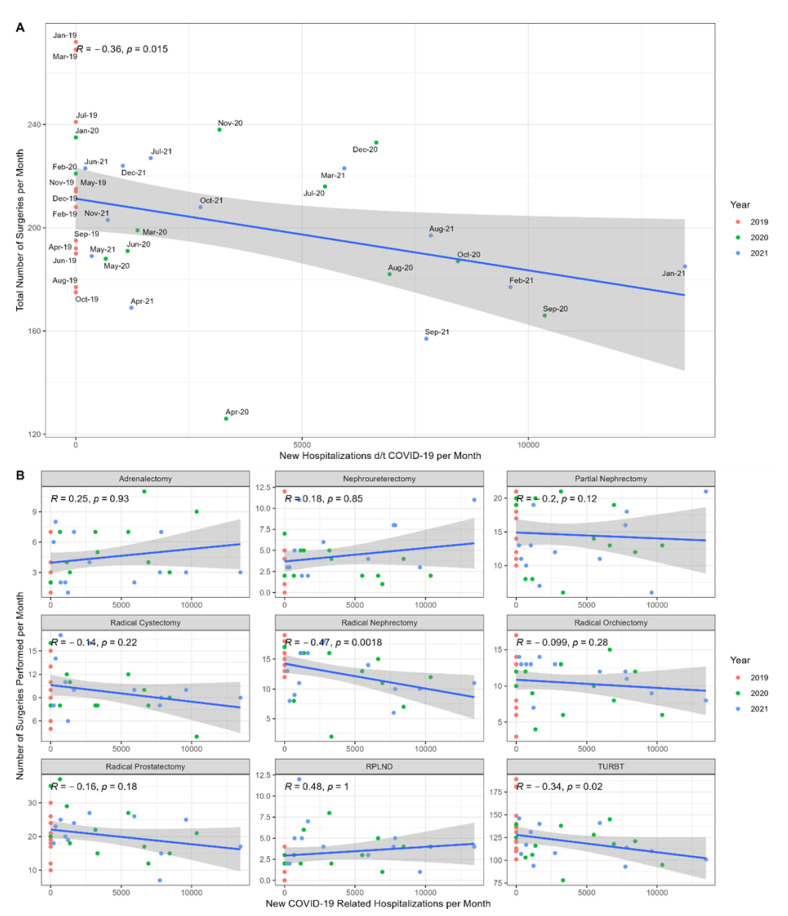
(**A**) Correlation between the total number of urologic surgeries per month and the number of new COVID-19 related hospitalizations per month. (**B**) Subgroup analysis per surgery type.

Our expectations that greater numbers of COVID-19-related hospitalizations would have a negative effect on urologic oncology surgical volumes were borne out. There was a significant negative correlation between the total number of surgeries performed per month and the number of new COVID-19-related hospitalizations (*R* = −0.36, *p* = 0.015) (Figure 3). The results of a subgroup analysis per surgery type demonstrated a significant negative correlation for radical nephrectomies (*R* = −0.47, *p* = 0.0018) and TURBTs (*R* = −0.34, *p* = 0.02).

### 3.4. Correlations between the Average Monthly OSI in Israel and Monthly Urologic Oncology Surgical Volumes

**Figure 4:**
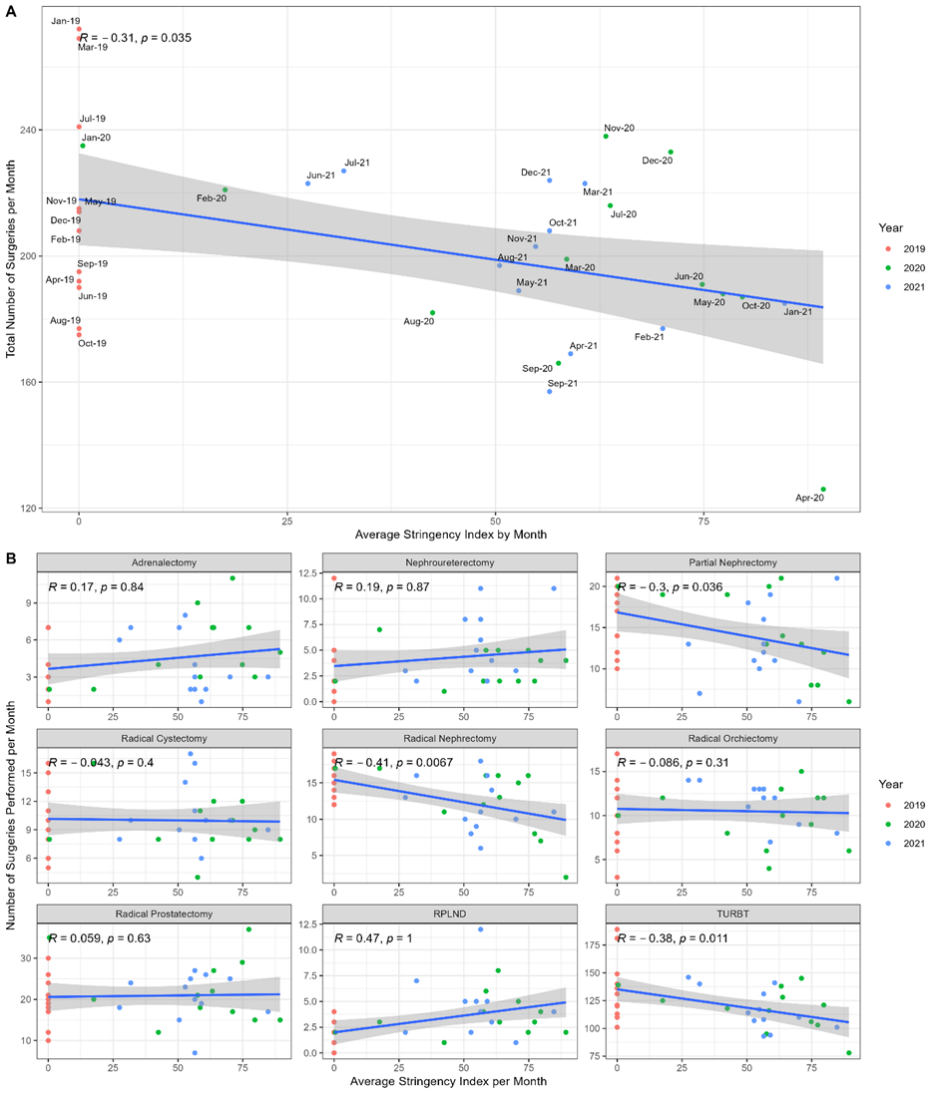
(**A**) Correlation between the total number of urologic surgeries per month and the monthly average Oxford Stringency Index. (**B**) Subgroup analysis per surgery type.

A significant negative correlation was demonstrated between the total number of surgeries performed per month and the monthly average OSI values (*R* = −0.31, *p* = 0.035) (Figure 4). A subgroup analysis per surgery type demonstrated a significant negative correlation for radical nephrectomies (*R* = −0.41, *p* = 0.0067), partial nephrectomies (*R* = −0.3, *p* = 0.036), and TURBTs (*R* = −0.38, *p* = 0.011).

### 3.5. Changes in Urologic Oncology Surgery Volumes in Relation to COVID-19 Wave Progression

**Figure 5:**
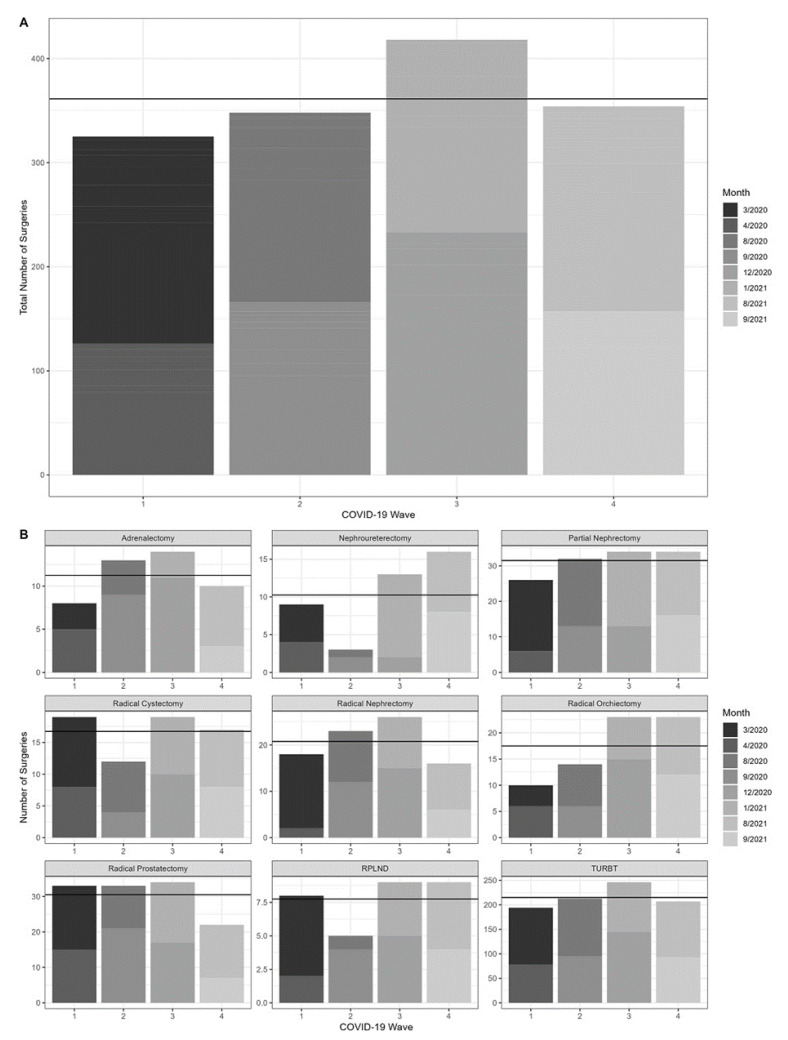
(**A**,**B**) Monthly urologic surgery volumes per COVID-19 wave and subgroup analysis by surgery type. The horizontal black line indicates the overall mean number of surgeries.

In order to examine changes in the healthcare response to progression of the COVID-19 pandemic, we opted to analyze changes in urologic oncology surgery volumes per COVID-19 wave. As demonstrated in Figure 5, there was a general positive trend, with surgical volumes increasing with each wave ($X^{2}$ = 13.184, *df* = 3, *p* = 0.004). A subgroup analysis per surgery type showed a statistically significant positive trend for nephroureterectomies ($X^{2}$ = 9.244, *df* = 3, *p* = 0.026) and near-significant results for radical orchiectomies ($X^{2}$ = 7.371, *df* = 3, *p* = 0.06) and TURBTs ($X^{2}$ = 6.837, *df* = 3, *p* = 0.077). Since the data on the fifth COVID-19 wave (Omicron variant) were incomplete, the surgery volumes during December 2021 were not included.

### 3.6. Correlations between the Cumulative First-Dose Vaccination Rate in Israel and the Monthly Surgical Volumes

**Figure 6:**
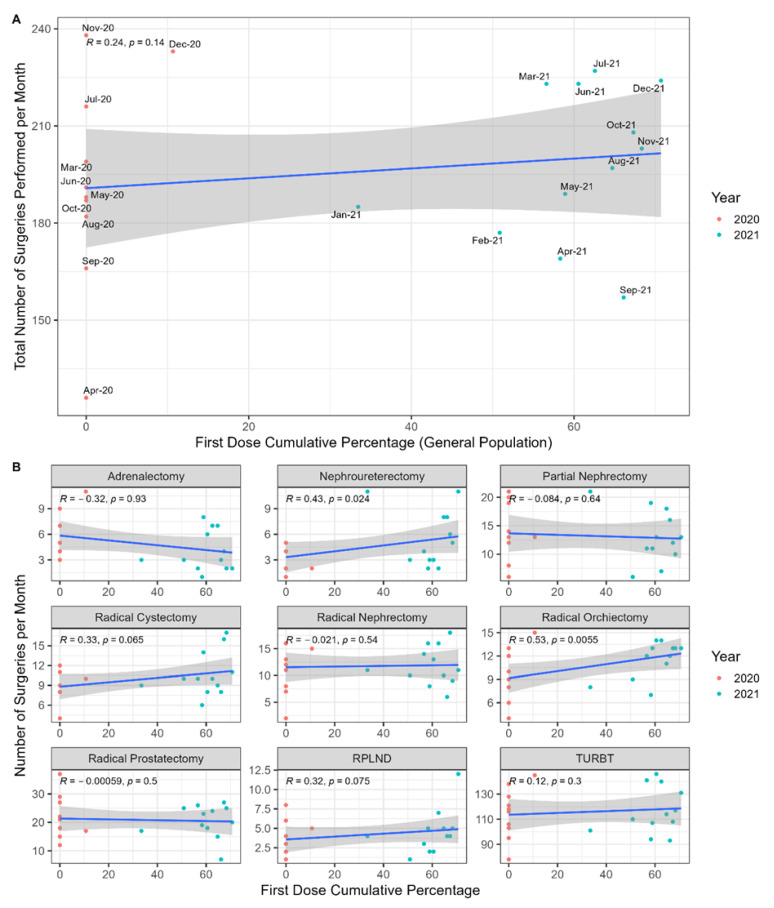
(**A**) Correlation between the first vaccination dose and the total number of urologic surgeries per month. (**B**) Subgroup analysis per surgery type.

We hypothesized that higher vaccination rates would correlate positively with urologic oncology surgical volumes, but there was no significant correlation between the total number of surgeries performed per month and the cumulative first dose vaccination percentages (Figure 6). However, the numbers of radical orchiectomies and nephroureterectomies did show significant positive correlations to vaccination rates (*R* = 0.53, *p* = 0.0055 and *R* = 0.43, *p* = 0.024, respectively).

## 4. Discussion

We present novel evidence of an accommodation of urologic oncology surgical volumes to the imposition of restraints of the healthcare system in response to the COVID-19 pandemic. Our results showed a significant rising trend in surgical volumes with each subsequent COVID-19 wave during the study period. We were also able to show that these surgical volumes correlated negatively with COVID-19-related hospitalizations and containment measures. Subgroup analysis per urologic surgery type revealed that these correlations stemmed from and could be attributed to changes in TURBT and radical nephrectomy volumes as well as to partial nephrectomies. Given that TURBTs comprised the majority of all urologic oncology surgery procedures in our study (4415/7327, 60.25%), this may account for changes in statistical significance as well as correlation coefficients among pandemic-related parameters. It is worth noting that the number of COVID-19 cases did not correlate significantly with urologic oncology surgery volumes. This can be partly explained by the mixed trends observed in the subgroup analysis of each surgery type. Additionally, the number of COVID-19 cases in our analysis may be prone to bias, since the availability of COVID-19 testing modalities varied greatly throughout the study period [16]. Interestingly, the cumulative first vaccine dose percentages did not correlate significantly with the total number of surgeries performed per month. However, the subgroup analysis per surgery type revealed statistically significant positive correlations for the radical orchiectomy and nephroureterectomy subpopulations. These results are consistent with our demonstration of a trend towards rising urologic surgery volumes with subsequent pandemic waves. Several studies examined the effects of the COVID-19 pandemic in the specialty of urologic oncology surgery and surgical oncology in general [8,17]. Stöss et al. [5] reported an average reduction of 29.3% in oncological resections, as well as reductions in hospital bed and operating room capacities throughout Europe. Similar findings were found by Guerrieri et al. [18] who cited a 26.7% reduction in urologic oncology surgeries between 2019 and 2020 in Italy. In another study performed by the COVIDSurg Collaborative [14] that covered 61 countries, 10% of eligible patients did not undergo surgery during the study follow-up period, all due to COVID-19-related reasons for non-operation. Other findings of that study included a rise in non-operation rates compatible with lockdown stringency. Those data are consistent with our findings on inverse correlations between urologic oncology surgery volumes, COVID-19-related hospitalizations, and the OSI as surrogates of the burden on the Israeli healthcare system and on the government and public health policies, respectively. Importantly, data on hospitalizations are less prone to bias with regard to the availability of COVID-19 screening modalities and serve as a more reliable surrogate for disease burden on Israeli healthcare facilities than COVID-19 case numbers. Furthermore, these findings present strong internal validation in terms of data curation, as evident by the compatibility of the inspected parameters and the respective correlations. Indeed, public health policies emerged as having a direct and significant effect upon surgery volumes, including oncological procedures. For several urologic malignancies, surgical delay has been shown to affect patient prognosis detrimentally; This is especially apparent for high-priority diseases, such as muscle invasive bladder cancer (MIBC), high-grade upper tract urothelial carcinoma (UTUC), large renal masses (T3 or higher), testicular and penile cancer. In contrast, it should be highlighted that not all delays have a significant negative impact on patient prognosis; For instance, small renal masses (<4 cm) and very low-low risk prostate cancer can safely undergo active surveillance [19]. Numerous studies [7,20,21,22] have demonstrated the effect of strict COVID-19-related healthcare measures and the resulting surgical delays on urologic malignancy progression and cure rates. For example, Tulchiner et al. [20] showed a significant increase of high-grade tumors and advanced tumor stages in 2020 in comparison to 2019, in conjunction with fewer endoscopic procedures performed during the first 6 months of 2020 in one Austrian center. The findings of our study highlight the necessity for the formulation of adaptable public health policies which may be implemented during future pandemics as well. In this context, it is worth noting that the Oxford Stringency Index enables both quantification and evaluation of the effects of public health measures on urologic oncological procedures as a surrogate for oncological morbidity in general. Both caregivers and patients adapted well to the new reality dictated by the pandemic. For example, our analysis showed a significant inverse correlation between the OSI and the number of partial nephrectomies. One possible explanation is that surgeons were more likely to postpone intervention for low-risk disease, such as small renal masses, as demonstrated by Rosenzweig et al., and Guerrieri et al. [8,18]. Conversely, we also found a negative correlation between radical nephrectomies and the OSI, indicating maladaptation of the Israeli healthcare system to the needs arising from the COVID-19 pandemic, especially those regarding urologic oncological surgery, given that surgical delays for large tumors had been found to be associated with worse outcomes [23]. We also found such correlations in relation to the number of TURBT procedures, but the effect on patient prognosis could not be properly assessed since some of these procedures are performed for low-risk non-muscle-invasive bladder cancer for which a delay in treatment will not necessarily result in adverse outcomes. In contrast, delays in diagnosis were shown to be linked with worse prognosis for muscle-invasive bladder cancer, [24], as were longer periods of time between diagnosis and radical cystectomy [25]. Interestingly, although we were able to demonstrate an adaptation of the healthcare system (Section 3.5) to the COVID-19 pandemic restrictions with regard to enhanced urologic oncology surgery volume, the effect of public health measures and hospitalization burden on these procedures was still significant (Section 3.3 and Section 3.4). Notwithstanding, it appears that some procedures, such as radical prostatectomies, were not affected by either the adaptation to the setting of this pandemic healthcare policies or disease burden, as evident by non-significant correlations with either COVID-19-related hospitalizations, the Oxford Stringency Index or vaccination rates. Our main aim of this study was to assess the effect of vaccination campaigns on the acclimation process of healthcare systems with regards to urologic oncology surgery. With Israel spearheading both vaccination campaigns and related research, with implications on a national and global scale [11,12,26,27], we believe that our study presents a highly suitable model for inspecting the influence of vaccination rates on the field of urologic oncology surgery. However, while the surrogates of COVID-19-related hospitalizations and OSI were found to be correlated to trends in total surgical volumes, no similar link was found for the cumulative vaccination rates. It is possible, however, that vaccination rates may influence surgical volumes indirectly, as suggested by their effects on both government policies and disease burden. As shown in Figure 5 and Figure 6, both radical orchiectomies and nephroureterectomies, which have been associated with an increase in surgical volumes with subsequent pandemic waves, correlated positively with cumulative vaccination rates. Importantly, the correlation between vaccination status and radical orchiectomies may be related to this subpopulation’s young age. Although younger people are apparently less prone to developing severe COVID-19 [28], we speculate that different behavioral patterns compared to older patient subpopulations (e.g., tendencies of younger patients to delay urgent or emergency care due to COVID-19-related concerns [29]) as well as diagnostic delays are responsible for the observed trend. This may be partly explained by the detrimental effect of COVID-19 restrictions on healthcare utilization, as observed in non-geriatric populations [30]. Our study has several limitations. First, the data were curated by searching for relevant ICD-10 codes, and possible misclassification and/or omission of performed surgeries may affect data accuracy. Second, our study does not account for potential confounders since correlations were made solely with variables of interest. However, the results on indices relating to disease burden and public health measures emerged as being consistent. Third, the lack of a uniform trend in subgroup analysis per surgery type indicates a wide variability between both patient and caregiver subpopulations: this was the result of our study having been based on data from multiple medical centers.

## 5. Conclusions

The volumes of urologic oncology surgery had been detrimentally affected during the initial COVID-19 pandemic waves in Israel due to strict containment measures, including travel restrictions, economic lockdowns, and stringent health-oriented measures. Our study findings demonstrate the acclimation of the Israeli healthcare system to the COVID-19 pandemic with regard to lessening the delay in performing urologic oncology procedures.

## Data Availability

The data presented in this study are available on request from the corresponding author.
